# Anabolic-androgenic steroids for patients with chronic obstructive pulmonary disease: A systematic review and meta-analysis

**DOI:** 10.3389/fmed.2022.915159

**Published:** 2022-09-06

**Authors:** Yahui Liu, Chunrong Huang, Juan Du, Gelei Lan, Xueqing Du, Yidan Sun, Guochao Shi

**Affiliations:** ^1^Department of Respiratory and Critical Care Medicine, Ruijin Hospital, Shanghai Jiao Tong University School of Medicine, Shanghai, China; ^2^Institute of Respiratory Diseases, Ruijin Hospital, Shanghai Jiao Tong University School of Medicine, Shanghai, China; ^3^Shanghai Key Laboratory of Emergency Prevention, Diagnosis and Treatment of Respiratory Infectious Diseases, Ruijin Hospital, Shanghai Jiao Tong University School of Medicine, Shanghai, China

**Keywords:** chronic obstructive pulmonary disease, anabolic-androgenic steroids, meta-analysis, systematic review, randomized controlled trials

## Abstract

**Background:**

Testosterone deficiency is common in chronic obstructive pulmonary disease (COPD) patients. There has been a growing interest in the potential use of anabolic-androgenic steroids (AASs) in patients with COPD recently. However, whether AASs could improve their clinical outcomes remains unknown.

**Methods:**

In order to explore the efficacy of AASs in patients with COPD, systematic search of MEDLINE, Embase, the Cochrane Library and ClinicalTrials.gov for randomized controlled trials (RCTs) of AASs for COPD published before March 17, 2022 was performed.

**Results:**

Data were extracted from 8 articles involving 520 participants. The median number of participants per study was 39.5 and the mean follow up was 14.2 weeks. As compared to the control group, AASs therapy could significantly improve body weight (weighted mean difference (WMD), 1.38 kg; 95% CI, 0.79 to 1.97 kg), fat-free mass (WMD, 1.56 kg; 95% CI, 0.94 to 2.18 kg) and peak workload (WMD, 6.89W; 95% CI, 3.97 to 9.81W) of COPD patients, but no improvements in spirometry indicators and six-minute walking distances (WMD, 16.88 m; 95%, −3.27 to 37.04 m). Based on the available research data, it is uncertain whether AASs treatment could improve the quality of life of COPD patients.

**Conclusions:**

Limited published evidence indicates that AASs therapy provides clinical benefits in patients with COPD. However, longer and larger studies are needed to better clarify the efficacy of AASs and draw final conclusions.

## Introduction

Chronic obstructive pulmonary disease (COPD) is a globally prevalent illness, which affects millions of people and becomes the fourth leading cause of death in the world (1). The Global Burden of Disease study estimated that 174.5 million adults worldwide had prevalent COPD and 3.2 million deaths were estimated to be due to COPD in 2015 (2, 3). The burden of COPD is expected to increase in coming decades by reason of aging of the population and continued exposure to COPD risk factors (4). Additionally, COPD often coexists with other diseases (comorbidities) that may influence the disease course. Many people suffer from this disease for years and die prematurely from it or its comorbidities. Involuntary weight loss, decreased muscle function and impaired exercise capacity are common comorbidities of COPD, which are associated with poor health status and prognosis (1). Comorbidities like these remain largely under-recognized and underdiagnosed, especially in low-income and middle-income countries (5). In the meantime, medical treatment is predominantly focused on the primary organ dysfunction. However, treatment of these comorbidities is of great importance, as they are potentially remediable.

Androgens belong to a class of steroid hormones. Testosterone (TT) and dehydroepiandrosterone (DHEA) are the principal circulating androgens. Anabolic-androgenic steroids (AASs) are synthetic derivatives of TT that were originally developed as adjunct therapy for a variety of medical conditions. In the past few decades, various studies have demonstrated that androgens could exert anti-inflammatory and protective effects through direct or indirect effects in pulmonary diseases (6–8). Several randomized controlled trials (RCTs) investigated the influence of exogenous androgen therapy on body composition, muscle strength, exercise capacity and health-related quality of life (HRQoL) in patients with COPD (9–19).

Atlantis et al. included six RCTs about TT supplementation in COPD for meta-analysis (8). However, they only focused on exercise capacity and HRQoL, and did not pay attention to spirometry and body composition (8). Pan et al. reperformed meta-analysis on this issue including eight RCTs. TT or androgen derivative treatment was the trial arm of six RCTs, and the other two involved recombinant human growth hormone and ghrelin, which greatly affected our confidence in the certainty of evidence (20). More importantly, two recently published large-scale clinical studies have demonstrated that higher levels of TT are associated with better lung function in men, and low dehydroisoandrosterone sulfate (DHEA-S) levels in women were associated with impaired lung function and a greater risk of developing airflow limitation later in adult life (21, 22). Additionally, Baillargeon et al. conducted two retrospective cohort studies, and found that TT replacement therapy may slow disease progression and decrease hospitalization rate in patients with COPD (23). Thus, we believe that studies on the efficacy of AASs therapy for COPD are far from over. In the light of the above considerations, we conduct a meta-analysis of RCTs to evaluate the potential benefits of exogenous AASs therapy on COPD patients, mainly concentrating on body composition, lung function, exercise capacity and HRQoL, hoping to call on more researchers to pay attention to this kind of therapy.

## Methods

### Search strategy and study selection

Two reviewers performed a comprehensive literature search for RCTs evaluating the effects of AASs in patients with COPD. We employed a highly sensitive search strategy to retrieve articles and supplementary materials contain the search strategies developed for MEDLINE database interfaces, which we adapted to search other databases. Databases to search in retrieving relevant papers included the following: MEDLINE, Embase, the Cochrane Library and ClinicalTrials.gov. The databases were searched for studies published before March 17, 2022. Furthermore, we reviewed citations in the retrieved articles to search for additional relevant studies.

### Criteria for considering studies for this review

For evidence on the effectiveness of AASs for the treatment of COPD, we considered only RCTs written in English. Reviews and animal studies were excluded. Studies that were not published as full reports were not included, either. The most comprehensive publication was used when there were several involving the same study population. Two reviewers independently checked the relevant studies obtained from databases. Any difference in opinion about eligibility was resolved by consensus.

Inclusion criteria were: (1) population: adults (≥18 years), diagnosed with COPD, (2) intervention: using AASs defined as receiving oral or intramuscular injection of TT, DHEA, nandrolone, oxymetholone, dihydrotestosterone (DHT), oxandrolone or anabolic steroids, (3) outcomes: refer to “type of outcome measures.” Based on the searched results, we selected the primary outcomes to be: improvements in lung function, body composition, and exercise capacity from baseline to follow up in subjects treated with AASs. The secondary outcomes were the changes of HRQoL.

### Data extraction

Data from included RCTs were independently extracted by two investigators and checked by other authors in agreement with Data Extraction for Complex Meta-Analysis recommendations (24, 25). The concordance rate was 91% between the two authors. Discrepancies in data extraction were resolved by discussion or arbitration by a third reviewer if agreement could not be reached. The following information was abstracted: authors, publication year, participant inclusion and exclusion criteria, sample size, duration of treatment, geographic locale in which the study took place, mean or median participant age, route of medication, dosage of administration, outcomes and adverse events.

### Assessment of study quality

The Cochrane risk-of-bias tool was used to assess the quality of the RCTs concerning on selection bias, performance bias, detection bias, attrition bias, reporting bias and other bias. Two reviewers independently assessed the quality of individual studies, and any difference in opinion about the quality score was resolved by consensus. The degree of bias found in the individual studies were categorized into high, moderate, or low risk of bias according to the Cochrane risk of bias tool.

### Date synthesis and analysis

The meta-analysis was conducted using Review Manager 5.4 (Cochrane Collaboration, London, England). The weighted mean differences (WMD) and 95% confidence interval (CI) measured prior to initiating and then after treatment with either exogenous androgens or placebo were calculated for each individual study. The standardized mean differences (SMD) and 95% CI were applied between the two groups when the studies all assessed the same outcome but measured it in a variety of ways (for example, some studies measured HRQoL, but they use different scales). If the associated information was present merely in figures in eligible studies, three investigators would use Engauge Digitizer 12.1 to collect data from the statistical graphs independently. Then, the mean values would be adopted. Q statistic and *I*^2^ index were used to examine statistical heterogeneity. Moderate to high levels of heterogeneity were considered for *P*_*heterogeneity*_ <0.10 or *I*^2^ > 50%. Random effects meta-analysis was used for high between-study heterogeneity. Publication bias was evaluated by funnel plots. Sensitivity and subgroup analysis were conducted to determine the robustness of the pooled results and to explore the possible source of heterogeneity. Statistical tests were two-sided and used a significance threshold of *P* < 0.05.

## Results

### Study characteristics and risk of bias

**Figure 1:**
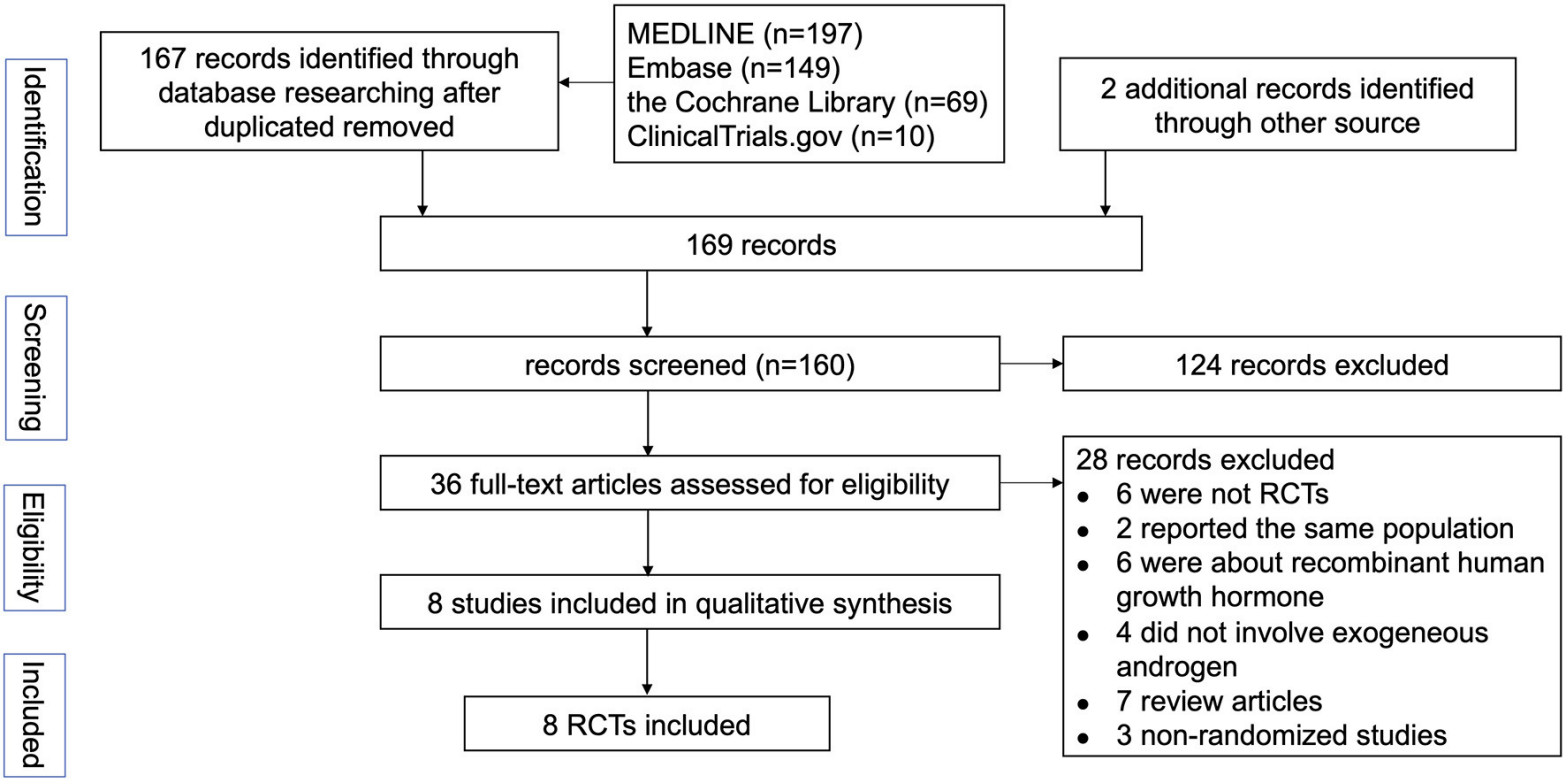
Flow diagram for study selection. RCTs, randomized control trials.

The selection process for the studies included in the meta-analysis was outlined in Figure 1. Eight RCTs involving 520 patients with COPD were included in this meta-analysis (Table 1). The median number of participants was 39.5 (range, 16–203), and the mean duration of follow-up was 14.2 weeks. Two studies took place in the Netherland, one in Brazil, one in Norway, one in the United Stated of America, one in Canada, one in France and one in India. Five studies recruited only male patients as subjects. The detailed inclusion and exclusion criteria presented in Supplementary Table S1. Most of the studies investigated body composition, exercise capacity and HRQoL as outcomes. All studies reported the treatment-related adverse events. If any, the numbers and reasons of withdrawals were also mentioned. Venous blood concentration changes of sex hormone were measured in five trials. Two studies did not specify how serum testosterone levels were measured.

**Table 1:** Selected characteristics of the eight RCTs included in this systematic review.

**Authors**	**Year of publication**	**Area**	**Number of randomized populations**	**Per-protocol population**	**Sex (M/W)**	**Mean age (years) Experimental/ Control**	**Types of androgens and dosage**	**Grouping**	**Duration of treatment**	**Safety measures**
Schols et al.	1995	The Netherlands	217	203	M/W	NR	ND on day 1, 15, 29 and 43	P: placebo N: placebo + nutrition N+A: ND + nutrition	8 weeks	no
Ferreira et al.	1998	Brazil	23	17	M	70.3/66.1	testosterone	placebo group and testosterone group	27 weeks	no
Creutzberg et al.	2003	The Netherlands	63	56	M	66/67	50 mg ND on day 1, 15, 29 and 43	placebo group and ND group	8 weeks	ESR declined. LDH elevated.
Svartberg et al.	2004	Norway	29	27	M	64.5/67.5	250 mg testosterone every fourth week	placebo group and testosterone group	26 weeks	no
Casaburi et al.	2004	USA	53	47	M	No training: 66.6/67.6 Training: 66.4/68.9	100 mg/week of testosterone	placebo; testosterone; placebo + training; testosterone + training.	10 weeks	Hemoglobin elevated
Sharma et al.	2008	Canada	16	16	M/W	71.0/64.2	50 mg testosterone biweekly for men and 25 mg for women	Placebo group and ND group	16 weeks	no
Pison et al.	2011	France	126	122	M/W	66.6/65.1	oral testosterone undecanoate, M 80 mg/W 40 mg twice daily with PR	Control group and multimodal+ nutritional+ rehabilitation group	90 days	no
Daga et al.	2014	India	32	32	M	60.05/56.75	25 mg ND on days 1, 8, 15, 22, 29, and 35	placebo group and ND group	6 weeks	no

RCTs, randomized controlled trials; W, women; M, man; NR, not reported; ND, nandrolone decanoate; P, placebo; N, nutrition supplementation; PR, pulmonary rehabilitation; ESR, erythrocyte sedimentation rate; LDH, lactate dehydrogenase.

**Figure 2:**
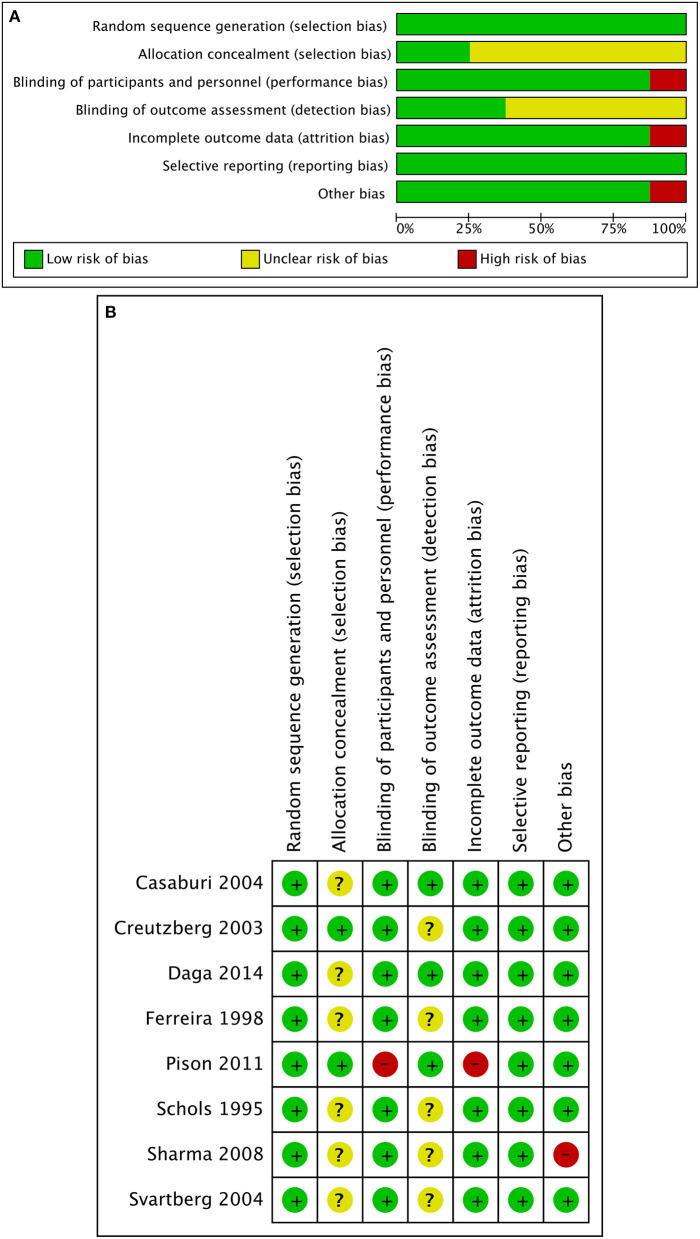
Quality assessment of included randomized control trials. **(A)** Risk of bias graph judging by Cochrane risk-of-bias tool. **(B)** Risk of bias summary. Insufficient details were reported about allocation concealment of the studies by Casaburi 2004 and Daga 2014. Insufficient details were reported about allocation concealment and outcome assessors of the studies by Ferreira 1998, Schols 1995, and Svartberg 2004. Insufficient details were reported about outcome assessors of the study by Creutzberg 2003. Blinding can be difficult, and the rate of lost to follow-up was more than 10% in the study by Pison 2011. Insufficient details were reported about allocation concealment and outcome assessors of the study by Sharma 2008, and the study was discontinued following the interim analysis.

The majority of studies were found to be at low risk of bias (Figure 2). However, insufficient details were reported about allocation concealment and outcome assessors in the study by Sharma et al. which was discontinued following the interim analysis (15). In addition, according to the experimental design by Pison et al., blinding during the trial can be difficult or even impossible, and the rate of lost to follow-up was more than 10% (16).

### Meta-analysis results

#### Effects of AASs on body composition

**Figure 3:**
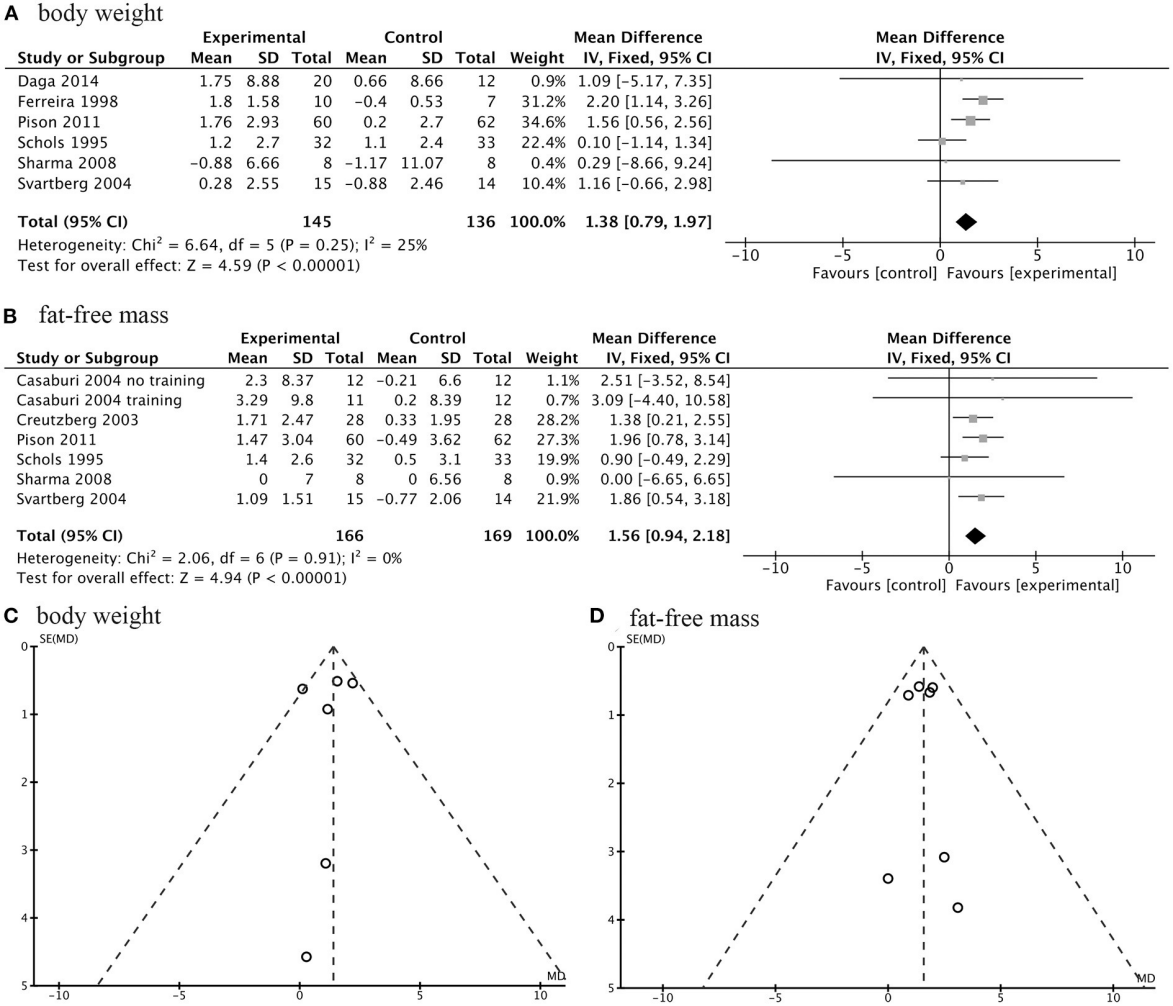
Forest plots of meta-analysis of body composition and publication bias assessed by funnel plot. Fixed-effects meta-analysis of effectiveness of AASs on body weight **(A)** and fat-free mass (FFM) **(B)** of COPD. Funnel plot of meta-analysis on body weight **(C)** and FFM **(D)**.

There were seven studies with a total number of 335 participants investigating the effects of AASs on changes in body composition of COPD patients. The meta-analysis indicated that exogenous AASs therapy significantly improved body weight (WMD, 1.38 kg; 95% CI, 0.79 to 1.97 kg; *I*^2^, 25%; *P* < 0.001; Figure 3A) and fat-free mass (FFM) (WMD, 1.56 kg; 95% CI, 0.94 to 2.18 kg; *I*^2^, 0%; *P* < 0.001; Figure 3B). In addition, funnel plots were produced which showed only slight evidence of publication bias for body weight and FFM (Figure 3). There was no statistically significant difference in pooled changes in midarm circumference (WMD, 0.36 cm; 95% CI, −0.33 to 1.04cm; *I*^2^, 20%; *P* =0.31) and percentage of body fat mass (WMD, −1.14%; 95% CI, −2.56 to 0.28%; *I*^2^, 0%; *P* =0.12) from baseline to follow up in COPD patients treated with AASs compared with those receiving placebo.

#### Effects of AASs on spirometry and exercise capacity

To measure effects of AASs on spirometry of individuals with COPD, several studies were involved in this meta-analysis. No significant differences were found on the improvements of maximal inspiratory muscle strength, peak oxygen uptake, predicted forced expiratory volume in the first second (FEV1 % pred), PaO2, and PaCO2 (Table 2).

**Table 2:** The results of pooled meta-analysis on spirometry and exercise capacity.

	**No. studies**	**Heterogeneity**		**Effect measure**	**WMD (95% CI)/ SMD (95% CI)**
		***P_*heterogeneity*_***	***I^2^*, %**	**Random effects/Fixed effects**	
**Spirometry**					
FEV_1_, % pred	3	0.87	0	fixed	−1.61 (−7.07, 3.84)^*^
PaO_2_, mmHg	4	0.72	0	fixed	0.52 (−3.07, 4.10)^*^
PaCO_2_, mmHg	3	0.74	0	fixed	−1.4 (−4.15, 1.35)^*^
**Exercise capacity**					
maximal inspiratory muscle strength	4	0.61	0	fixed	0.23 (−0.11, 0.57)^†^
peak oxygen consumption	4	0.43	0	fixed	0.07 (−0.27, 0.41)^†^
peak workload	5	0.49	0	fixed	6.89 (3.97, 9.81)^*^
6MWD	5	0.21	31	fixed	16.88 (−3.27, 37.04)^*^

FEV_1_, % pred, predicted forced expiratory volume in the first second; 6MWD, 6–min walking distances; CI, confidence interval; WMD, weighted mean difference; SMD, standardized mean difference.

^†^SMD (95% CI).

*WMD (95% CI).

**Figure 4:**
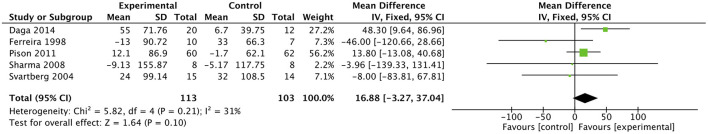
Forest plots of meta-analysis of 6-min walking distances.

Exercise capacity of COPD patients in these studies included were assessed using maximal inspiratory muscle strength, peak oxygen consumption, peak workload and 6-min walking distances (6MWD). The treatment of AASs to COPD patients could not improve their maximal inspiratory muscle strength and peak oxygen consumption, but increase the peak workload (WMD, 6.89W; 95% CI, 3.97 to 9.81W; *I*^2^, 0%; *P* = 0.49) (Table 2). There were five studies with a number of 213 participants investigating the effects of AASs on 6MWD. Our results showed no statistically significant difference in 6MWD between the two groups (WMD, 16.88 m; 95%, −3.27 to 37.04 m; *I*^2^, 31%; *P* = 0.21; Figure 4).

#### Effects of AASs on HRQoL

**Figure 5:**
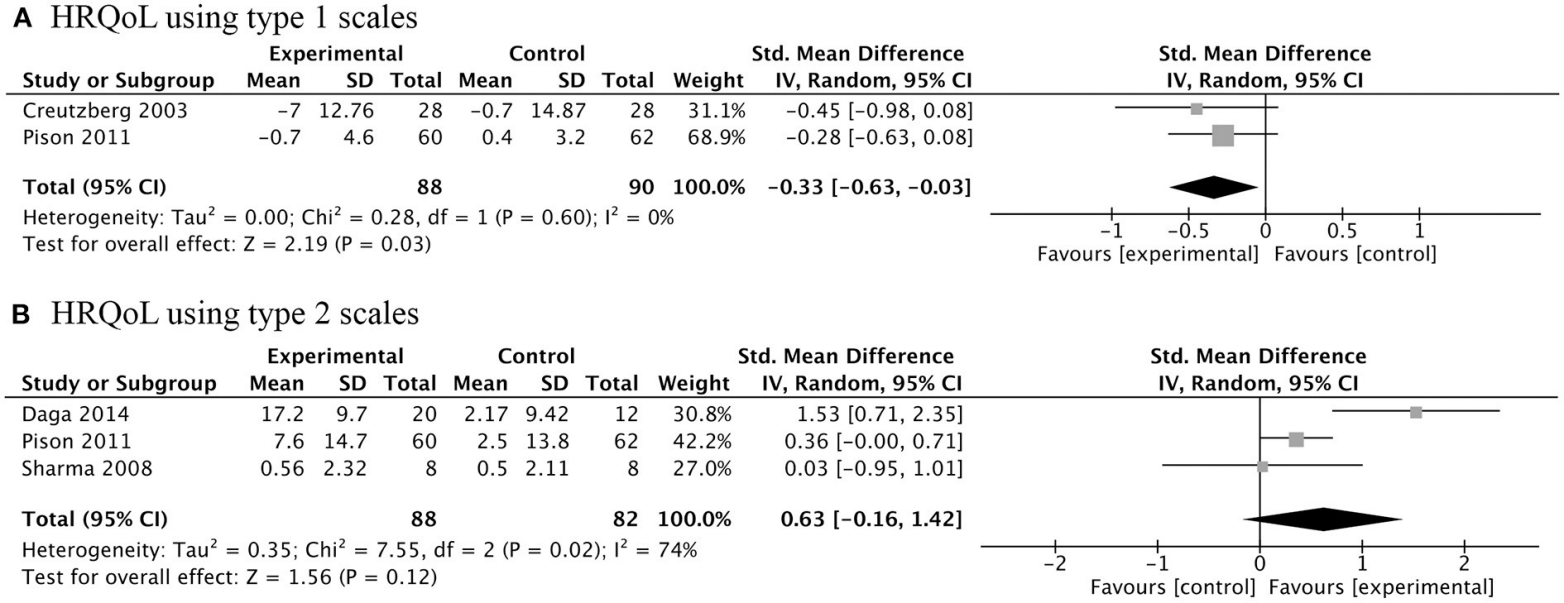
Forest plots of meta-analysis of health-related quality of life (HRQoL). Random-effects meta-analysis using type 1 scales **(A)** and type 2 scales **(B)** of effectiveness of AASs on HRQoL of COPD. Type 1 scales: Creutzberg 2003, St George's respiratory questionnaire; Pison 2011, Maugeri Foundation Respiratory Failure questionnaire. Type 2 scales: Daga 2014, Seattle Obstructive Lung Disease Questionnaire; Pison 2011, chronic respiratory disease questionnaire; Sharma 2008, chronic respiratory disease questionnaire.

Four RCTs investigated the effects of AASs therapy on COPD patients' HRQoL. There were two types of criteria used for judging HRQoL: one was that the higher the score, the higher the quality of life, including chronic respiratory disease questionnaire (CRQ) and HRQoL index (assessed by Seattle Obstructive Lung Disease Questionnaire); the other was just the opposite, including St George's respiratory questionnaire (SGRQ) and Maugeri Foundation Respiratory Failure questionnaire (MRF-28). Meta-analysis of the former type of scales showed that the treatment of AASs did not improve HRQoL of COPD participants (Figure 5A). However, the results of the latter types did not result in the same conclusion (Figure 5B). This means that we need more high-quality clinical trials to clarify this issue.

#### Effects of AASs on biochemical indicators

**Figure 6:**
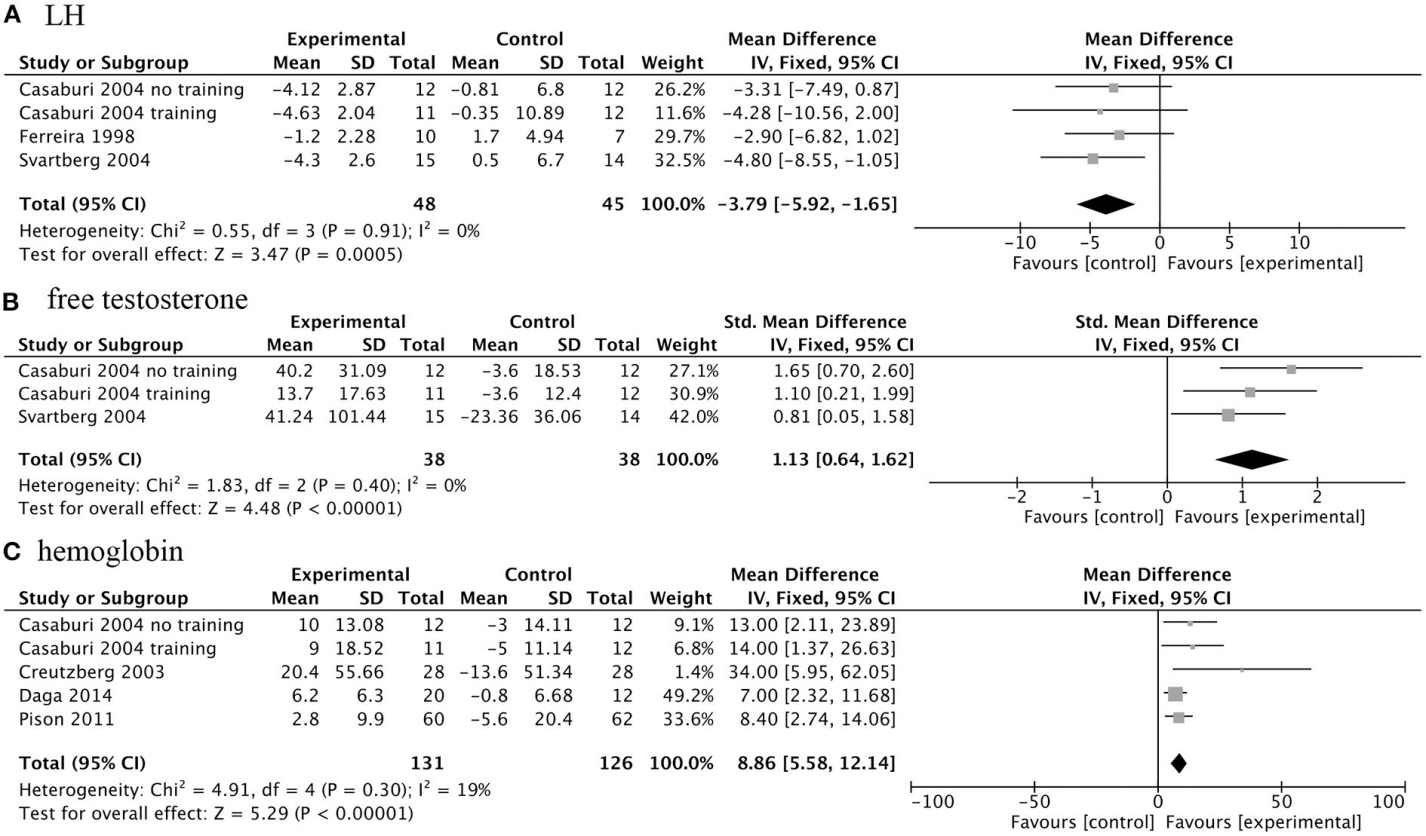
Forest plots of meta-analysis of biochemical indicators. Fixed-effects meta-analysis of effectiveness of AASs on luteinizing hormone (LH) **(A)**, free testosterone concentration **(B)** and hemoglobin **(C)** of COPD.

While improving some clinical outcomes, AASs for COPD patients will also cause changes in a series of biochemical indicators. Treatment of AASs could significantly decrease serum levels of luteinizing hormone (LH) (WMD, −3.79IU/L; 95% CI, −5.92 to −1.65 IU/L; *I*^2^, 0%; *P* < 0.001), and increased serum free testosterone concentration (SMD, 1.13; 95% CI, 0.64 to 1.62; *I*^2^, 0%; *P* < 0.001) and hemoglobin levels (WMD 8.86 g/L; 95% CI, 5.58 to 12.14 g/L; *I*^2^, 19%; *P* < 0.001) in COPD patients (Figure 6).

### Sensitivity analysis

The influence of individual studies on the overall summary estimates was examined by serially excluding each study in a sensitivity analysis. The pooled WMDs or SMDs for most of the outcomes were robust, except for peak workload and 6MWD. As shown in Table 3, the pooled WMD for peak workload changed after exclusion of one study which recruited malnourished patients with chronic respiratory failure, including not only COPD but also bronchiectasis, restrictive disorders and mixed respiratory disorders (16). As for 6MWD, three studies enrolled only male participants, but different route of medication and dosage of administration were used (10, 13, 18). Experimental group of one study received multimodal nutritional rehabilitation combining health education, oral nutritional supplements, exercise and oral testosterone treatment (16). Therefore, it is difficult to perform subgroup analysis for 6MWD.

**Table 3:** Sensitivity analysis of included randomized controlled trials for the outcome of peak workload and 6MWD.

**Outcomes**		**Number of studies**	**WMD (95% CI)**	**Heterogeneity**
				***P_*heterogeneity*_***	***I^2^*, %**
Peak workload	Fixed effects model	5	6.89 (3.97, 9.81)	0.49	0
	Exclusion of Casaburi 2004 no training	4	7.12 (4.18, 10.06)	0.64	0
	Exclusion of Casaburi 2004 training	4	6.91 (3.98, 9.85)	0.34	11
	Exclusion of Creutaberg 2003	4	7.01 (4.03, 9.98)	0.36	8
	Exclusion of Piosn 2011	4	0.22 (−8.65, 9.09)	0.81	0
	Exclusion of Sharma 2008	4	7.26 (4.28, 10.25)	0.56	0
6MWD	Fixed effects model	5	16.88 (−3.27, 37.04)	0.21	31
	Exclusion of Dage 2014	4	5.16 (−18.47, 28.78)	0.51	0
	Exclusion of Ferreira 1998	4	21.83 (0.89, 42.76)	0.41	0
	Exclusion of Pison 2011	4	20.85 (−9.63, 51.32)	0.13	47
	Exclusion of Sharma 2008	4	17.36 (−3.03, 37.74)	0.13	48
	Exclusion of Svartberg 2004	4	18.78 (−2.13, 39.69)	0.15	44

WMD, weighted mean difference; CI, confidence interval; 6MWD, 6–min walking distance.

## Discussion

### Summary of the main results

Eight RCTs were included in this meta-analysis. Our results indicated that exogenous AASs therapy could improve body mass, FFM, peak workload of COPD patients, with no significant changes in spirometry. Simultaneously, the treatment of AASs on 6MWD and HRQoL of COPD patients still needs further research (Supplementary Table S2).

### Interpretation of the results

Weight loss and decreased muscle function are common systemic manifestations in COPD, portending negative outcomes independent of the degree of airflow limitation, which occurred in more than 20% of COPD patients (2, 26). Besides, studies have shown that parameters of body composition are associated with exercise capacity, disease severity, mortality, disease prognosis, and quality of life (2–4, 26, 27). Decreased muscle function is in part the result of involuntary weight loss and muscle wasting in patients with COPD. The prevalence of muscle dysfunction increased from 20% in clinically stable outpatients up to 35% in patients eligible for pulmonary rehabilitation (PR) (28, 29). As the disease progresses, weight loss and muscle dysfunction may cause damage to skeletal muscle, affecting not only respiratory musculature, but also making an impact on peripheral skeletal muscle function, leading to fatigue, progressively deteriorating dyspnea and impaired exercise capacity. Dyspnea, muscle dysfunction, and airflow limitation deserve the blame for impaired exercise capacity, which stops patients from attending PR to alleviates symptoms, improves (HRQoL), and reduces hospital admissions and mortality (30). Thus, patients with COPD are trapped in a vicious circle of unfavorable prognosis. In addition to this, increases in body weight and physical activity were shown to relief symptoms, improve HRQoL, and reduce mortality (27).

Previous studies demonstrated that testosterone therapy could increase bone mineral density, FFM and muscle strength, reduce whole body fat, and improve maximal voluntary strength and muscle in healthy young hypogonadal men (31). Androgens can act on a variety of cells to exert protective effects, which may help partially explain androgen therapy for COPD. Androgens can easily diffuse across cell membranes without the need for receptor, or bind to classical and non-classical androgen receptors (ARs) to mediate genomic and non-genomic androgen effects, respectively (32–34). The DNA binding-dependent actions of the AR promote cardiac growth, kidney hypertrophy, cortical bone growth and regulate trabecular bone architecture (35). In addition, androgens generate anabolic effects on carbohydrate metabolism and protein, maintain insulin sensitivity, and impact brain function and mood (34). Exogenous supplementation of androgens could alleviate pulmonary artery hypertension, increase serum insulin-like growth factor (IFG) 1 and IGF-binding protein-1, reverse the loss in diaphragm force-generating capacity, improve mitochondrial and muscle function, increase myosin expression and attenuate pulmonary epithelial inflammation in COPD mouse model or patients (36–42). RCTs in older men with hypogonadism showed that exogenous testosterone therapy consistently increased bone mineral density and decreased fat mass, but the effects on muscle strength, physical function, energy, and mood were variable (31).

We found that AASs treatment could improve body composition, including body weight and FFM. Our conclusions obtained were not exactly the same as the previous studies, because the researches included and statistical methods adopted were different from the previous analysis (20). Additionally, sensitivity analysis showed that these results were robust, and no obvious heterogeneity and publication bias were found. Schols et al. showed that nutritional intervention alone helped COPD patients gain total weight (9). Under a combination treatment of exogenous androgens and nutrition supplementation, the weight gain of COPD patients is mainly FFM, which will benefit patients even more.

FFM is significantly associated with muscle strength, spirometry, physical function, quality of life, and survival in patients with COPD (20). However, no improvements in maximal inspiratory muscle strength, peak oxygen uptake, FEV1 %predicted, PaO2 and PaCO2 were observed in our meta-analysis. It was reported that men with severe COPD had lower free testosterone levels, and free testosterone was positively and independently associated with forced vitality capacity and FEV1 (43). Our analysis indicated that AASs improved free testosterone levels, but there was no improvement in spirometry. Diversities in quality of trials and inclusion criteria and small sample size may partially explain this difference.

As for exercise capacity, others found that testosterone supplementation therapy significantly improved several exercise capacity outcomes, such as peak muscle strength and peak oxygen outcomes (8). Another analysis indicated that anabolic steroids administration increased maximal inspiratory pressure and maximal expiratory pressure (20). We found that exogenous AASs therapy could increase patients' peak workload, with uncertain roles in 6MWD. The results of all analyses are not that robust, and additional large-scale studies are needed to determine whether exercise capacity could be improved with exogenous AASs treatment in COPD patients.

Alantis et al. pooled four clinical trials and drew a conclusion that TT treatment failed to improve HRQoL, but they did not classify different scales of HRQoL (8). On the contrary, others found that anabolic steroid administration improved HRQoL as measured by SGRQ total score and symptoms score (20). It is worth noting that there were only two trials included in latter analysis, so the conclusion was not that convincing, not to mention that the two studies in this meta-analysis were not limited to the use of AASs. Given that different scales were used to assess patients' HRQoL in different RCTs, we classified them into two categories. Combined with our meta-analysis results and sensitivity analysis, we think additional trials must be conducted to assess the significance of this intervention.

Our meta-analysis shows that AASs therapy could significantly decrease serum LH, increase free testosterone and hemoglobin levels. We particularly emphasize that when applying exogenous AASs to treat patients with COPD, the changes of these treatment-related parameters need to be closely monitored.

### The implication for future studies

The therapeutic use of AASs in patients with chronic disease is appealing and the theoretic basis for it appears sound. However, the results from studies exploring the effects in patients with COPD are mixed, showing limited positive effects on muscle function or exercise capacity. There are lots of reasons why we believe it is premature to deny its potential efficacy. The definition of testosterone deficiency, dosages used for replacement (sub-physiological vs. supra-physiological), small trials with variable inclusion criteria and study populations varied widely across the studies.

In other clinical trials, doses up to 600 mg of testosterone per week for a healthy man were administered, which caused relatively few adverse events and improved muscle mass and strength in healthy men (44). It can be expected that the administered dose of exogenous AASs in the above-mentioned studies is probably too low to exert a clinically meaningful effect, since the highest dose of androgens applied in the eight trials was 250 mg (Table 1). For a clinically meaningful effect, higher doses of androgens maybe used, preferably in combination with PR and nutritional supplementation. Additionally, nandrolone is an anabolic steroid that cannot be converted to DHT, and is considered to be less androgenic than testosterone. Compared with testosterone, nandrolone may be expected to be better tolerated, especially in women. Therefore, we believe that it is suggesting that exogenous AASs therapy was likely to be an acceptable type of administration used in COPD patients, especially those with advanced COPD, involuntary weight loss, muscle wasting and on chronic corticosteroid therapy.

### Limitations

Some limitations of this review are summarized as follows. Only a small number of studies were included and the small sample size was also a major limitation. Gender composition was different in this meta-analysis. Three trials recruited both male and female subjects, and five trials included only men. The duration of treatment for these studies ranged from 6 weeks to 27 weeks. Four trials looked at the effect of exogenous testosterone, whereas others used nandrolone. In addition, there were also differences in the route of administration, the dosage of exogenous androgens, whether the intervention included nutritional supplements and exercise training or not. All of these may contribute to the risk of bias.

## Conclusion

In general, the main findings from our meta-analysis indicate that AASs therapy can increase body weight, FFM and peak workload in COPD patients. However, because of the limited number of included trials, we are not certain whether it has improvements on spirometry, 6MWD and HRQoL. More multi-center RCTs in the future are of great essence, especially these with higher quality and longer follow-up duration.

## Data availability statement

The original contributions presented in the study are included in the article/Supplementary material, further inquiries can be directed to the corresponding author.

## Author contributions

Conceived and designed the experiments: GS. Performed the experiments: YL, JD, CH, GL, and XD. Analyzed the data: YL, JD, and YS. Contributed reagents, materials and analysis tools: YL, CH, and GL. All authors contributed to drafting and revising the article, and gave final approval of the version to be published.

## Funding

This study was supported by Grant 22YF1424800 from Shanghai Sailing Program, Grant 82170023, 81970020 from National Natural Science Foundation of China, Grant 2019SY006 from Shanghai Municipal Health Commission, Grant 20dz2261100 from Shanghai Key Laboratory of Emergency Prevention, Diagnosis and Treatment of Respiratory Infectious Diseases, Grant shslczdzk02202 from Shanghai Municipal Key Clinical Specialty, Grant 20dz2210500 from Cultivation Project of Shanghai Major Infectious Disease Research Base, Grant 2017ZZ02014 from Shanghai key discipline for respiratory diseases.

## Conflict of interest

The authors declare that the research was conducted in the absence of any commercial or financial relationships that could be construed as a potential conflict of interest.

## Publisher's note

All claims expressed in this article are solely those of the authors and do not necessarily represent those of their affiliated organizations, or those of the publisher, the editors and the reviewers. Any product that may be evaluated in this article, or claim that may be made by its manufacturer, is not guaranteed or endorsed by the publisher.
