# Supplementary material for: Anabolic-androgenic steroids for patients with chronic obstructive pulmonary disease: A systematic review and meta-analysis
Source: Front Med (Lausanne). 2022 Sep 6;9:915159. doi: 10.3389/fmed.2022.915159 (PMC9485876; doi:10.3389/fmed.2022.915159)
Supplement: Supplementary file 1 [file Table_1.docx]

**Table S1. Selected characteristics of the eight RCTs included in this systematic review**

| Authors | Year of publication | Inclusion criteria | Exclusion criteria |
| --- | --- | --- | --- |
| Schols et al^1^ | 1995 | moderate to severe COPD, in a stable clinical condition | demonstrated an increase in FEV_1_ > 10% of the predicted baseline value after administration of a bronchodilating agonist (400 ug salbutamol), unstable COPD, obesity, malignancies, ischemic heart disease, or other cardiac impairment, renal, hepatic, gastrointestinal, or endocrine diseases, and a history of recent (< 2 months) surgery |
| Ferreira et al^2^ | 1998 | ambulatory male patients, stable COPD (no respiratory exacerbation for at least 6weeks), BMI below 20kg/m^2^, PImax below 60% of the predicted value, did not have any other associated medical conditions that might have influenced their weight or respiratory muscle function | prostatic diseases or known cardiac diseases |
| Creutzberg et al^3^ | 2003 | fulfilled the criteria for COPD according to the American Thoracic Society guidelines, the FEV_1_ had to be < 70% of the reference value, the increase in FEV_1_ after inhalation of a beta-2-agonist < 10% of the reference value, clinically stable condition (not suffering from a recent respiratory tract infection) | obesity (body mass index > 30), malignancies, cardiac failure, chronic hypoxemia at rest requiring continuous oxygen support (PaO_2_ < 7.3kPa), GI inflammatory disorders, and insulin-dependent diabetes mellitus |
| Svartberg et al^4^ | 2004 | ambulatory male patients from the pulmonary out-patients clinic, aged 54–75, with moderate to severe COPD (forced expiratory volume in 1s (FEV_1_) < 60% of predicted), in stable condition | asthma, malignancies, cardiac impairment or hepatic or endocrine disease |
| Casaburi et al^5^ | 2004 | stable COPD, age 55 to 80 years, FEV_1_ of 60% predicted or less, FEV_1_ to vital capacity ratio of 60% or less, screening serum testosterone was 400 ng/dl or less (in the lower range for healthy older men) | significant cardiovascular or orthopedic impairments, body weight of less than 75% or more than 130% of ideal, symptomatic benign prostatic hypertrophy, prostate cancer history, serum prostate specific antigen of more than 4μg/L, or hemoglobin of more than 16g/dL |
| Sharma et al^6^ | 2008 | clinically stable and severe COPD according to the GOLD (Global Initiative for Obstructive Lung Disease) criteria, forced expiratory volume in one second (FEV_1_) less than 50% predicted and FEV_1_/FVC (forced vital capacity) ratio of less than 0.7 | asthma, unstable COPD, obesity, malignancy, ischemic heart disease, benign prostatic hypertrophy or prostate carcinoma, other cardiac impairment, renal, hepatic, gastrointestinal, or endocrine disease, recent surgery ≤2 months |
| Pison et al^7^ | 2011 | age > 18 years, room air arterial oxygen tension (PaO_2_) ≤8 kPa at initiation of home treatment, LTOT and/or home mechanical ventilation history > 3 months, BMI ≤ 21 kg/m^2^ or FFMI measured by 50 kHz bioelectrical impedance analysis (BIA) < 25th percentile of predicted which corresponds to FFMI < 18 kg/m^2^ in men and < 15 kg/m^2^ in women, absence of exacerbation within 3 months before inclusion, written informed consent. | pulmonary hypertension, obstructive sleep apnea syndrome, neuromuscular diseases, cystic fibrosis, any condition compromising 6-month survival, serum alanine aminotransferase > 1.5ULN, hormone-dependent cancer, elevated prostate-specific antigen (PSA), inability to follow a rehabilitation programme and women of childbearing age |
| Daga et al^8^ | 2014 | moderate‐ to severe‐COPD, aged 35 years and older, were able to the perform the spirometry test, FEV_1_ of < 80% (or evidence of airway obstruction with reversibility < 12% on pulmonary function testing) and room air PaO_2_ > 55 mmHg | patients with COPD with acute exacerbation and with any chronic illness |

**Supplemental reference:**

1. Schols AMWJ, Soeters PB, Mostert R, Pluymers RJ, Wouters EFM. Physiologic effects of nutritional support and anabolic steroids in patients with chronic obstructive pulmonary disease: A placebo-controlled randomized trial. *Am J Respir Crit Care Med*. 1995;152(4 I):1268-1274. doi:10.1164/ajrccm.152.4.7551381

2. Ferreira IM, Verreschi IT, Nery LE, et al. The influence of 6 months of oral anabolic steroids on body mass and respiratory muscles in undernourished COPD patients. *Chest*. 1998;114(1):19-28. doi:10.1378/chest.114.1.19

3. Creutzberg EC, Wouters EFM, Mostert R, Pluymers RJ, Schols AMWJ. A Role for Anabolic Steroids in the Rehabilitation of Patients with COPD? A Double-Blind, Placebo-Controlled, Randomized Trial. *Chest*. 2003;124(5):1733-1742. doi:10.1378/chest.124.5.1733

4. Svartberg J, Aaseboø U, Hjalmarsen A, Sundsfjord J, Jorde R. Testosterone treatment improves body composition and sexual function in men with COPD, in a 6-month randomized controlled trial. *Respir Med*. 2004;98(9):906-913. doi:10.1016/j.rmed.2004.02.015

5. Casaburi R, Bhasin S, Cosentino L, et al. Effects of testosterone and resistance training in men with chronic obstructive pulmonary disease. *Am J Respir Crit Care Med*. 2004;170(8):870-878. doi:10.1164/rccm.200305-617OC

6. Sharma S, Arneja A, McLean L, et al. Anabolic steroids in COPD: A review and preliminary results of a randomized trial. *Chron Respir Dis*. 2008;5(3):169-176. doi:10.1177/1479972308092350

7. Pison CM, Cano NJ, Chérion C, et al. Multimodal nutritional rehabilitation improves clinical outcomes of malnourished patients with chronic respiratory failure: A randomised controlled trial. *Thorax*. 2011;66(11):953-960. doi:10.1136/thx.2010.154922

8. Daga MK, Khan NA, Malhotra V, Kumar S, Mawari G, Hira HS. Study of body composition, lung function, and quality of life following use of anabolic steroids in patients with chronic obstructive pulmonary disease. *Nutr Clin Pract*. 2014;29(2):238-245. doi:10.1177/0884533614522832
