# Supplementary material for: Anabolic-androgenic steroids for patients with chronic obstructive pulmonary disease: A systematic review and meta-analysis
Source: Front Med (Lausanne). 2022 Sep 6;9:915159. doi: 10.3389/fmed.2022.915159 (PMC9485876; doi:10.3389/fmed.2022.915159)
Supplement: Supplementary file 2 [file Table_2.docx]

**Table S2. Summary of findings.**

**Author(s):** Yahui Liu, Chunrong Huang, Juan Du, Gelei Lan, Xueqing Du, Yidan Sun, Guochao Shi

**Question:** Exogenous AASs compared to placebo for COPD

**Setting:** pulmonary rehabilitation center; outpatients; intervention delivered by telephone, or in home setting

| **Certainty assessment** | | | | | | | **№ of patients** | | **Effect** | | **Certainty** | **Importance** |
| --- | --- | --- | --- | --- | --- | --- | --- | --- | --- | --- | --- | --- |
| **№ of studies** | **Study design** | **Risk of bias** | **Inconsistency** | **Indirectness** | **Imprecision** | **Other considerations** | **exogenous AASs** | **placebo** | **Relative (95% CI)** | **Absolute (95% CI)** |  |  |
| **weight gain (follow-up: range 6 weeks to 27 weeks; assessed with: kg)** | | | | | | | | | | | | |
| 6 | randomised trials | serious^a^ | not serious | serious^b^ | serious^c^ | none | 145 | 136 | - | MD **1.38 kg higher** (0.79 higher to 1.97 higher) | ⨁◯◯◯ Very low | IMPORTANT |
| **fat-free mass changes (follow-up: range 8 weeks to 26 weeks)** | | | | | | | | | | | | |
| 6 | randomised trials | serious^d^ | not serious | serious^e^ | not serious^f^ | none | 166 | 169 | - | MD **1.86 kg higher** (0.94 higher to 2.18 higher) | ⨁⨁◯◯ Low | IMPORTANT |
| **FEV1%** | | | | | | | | | | | | |
| 3 | randomised trials | serious^g^ | not serious | serious^h^ | serious^i^ | none | 58 | 50 | - | MD **1.61 % lower** (7.07 lower to 3.84 higher) | ⨁◯◯◯ Very low | CRITICAL |
| **peak workload** | | | | | | | | | | | | |
| 4 | randomised trials | serious^j^ | not serious | serious^k^ | serious^l^ | none | 119 | 122 | - | MD **6.89 W higher** (3.97 higher to 9.81 higher) | ⨁◯◯◯ Very low | IMPORTANT |
| **six-minute-walk distance** | | | | | | | | | | | | |
| 5 | randomised trials | serious^m^ | not serious | serious^n^ | serious^o^ | none | 113 | 103 | - | MD **16.88 m higher** (3.27 lower to 37.04 higher) | ⨁◯◯◯ Very low | IMPORTANT |
| **health-related quality of life** | | | | | | | | | | | | |
| 5 | randomised trials | serious^p^ | not serious | serious^q^ | serious^r^ | none |  |  | not estimable |  | ⨁◯◯◯ Very low | IMPORTANT |

**CI:** confidence interval; **MD:** mean difference

#### Explanations

a. Downgraded by one level for study limitations (risk of performance and attrition bias; interim termination of clinical trials)

b. Interventions were inconsistent across studies.

c. The total number of cases included was less than the optimal information size.

d. Downgraded by one level for study limitations (risk of performance and attrition bias; interim termination of clinical trials)

e. Interventions and measurement methods used were inconsistent across studies.

f. The total number of cases included was less than the optimal information size.

g. Downgraded by one level for study limitations (risk of performance and attrition bias)

h. Interventions and measurement methods used were inconsistent across studies.

i. The total number of cases included was far less than the optimal information size.

j. Downgraded by one level for study limitations (risk of performance and attrition bias)

k. Interventions and measurement methods used were inconsistent across studies.

l. The total number of cases included was less than the optimal information size.

m. Downgraded by one level for study limitations (risk of performance and attrition bias; interim termination of clinical trials)

n. Interventions used was inconsistent across studies.

o. The total number of cases included was less than the optimal information size.

p. Downgraded by one level for study limitations (risk of performance and attrition bias; interim termination of clinical trials)

q. Interventions and measurement methods used were inconsistent across studies.

r. The total number of cases included was less than the optimal information size.
