# Supplementary material for: Anabolic-androgenic steroids for patients with chronic obstructive pulmonary disease: A systematic review and meta-analysis
Source: Front Med (Lausanne). 2022 Sep 6;9:915159. doi: 10.3389/fmed.2022.915159 (PMC9485876; doi:10.3389/fmed.2022.915159)
Supplement: Supplementary file 3 [file Data_Sheet_1.docx]

Supplementary Material

# Search strategy:

1.1 For MEDLINE

((((((((((((((((((((((Gonadal Steroid Hormones[Text Word]) OR Sex Steroid[Text Word]) OR Sex Hormone[Text Word]) OR Steroid Hormone[Text Word]) OR Gonadal Steroid[Text Word]) OR Androgen[Text Word]) OR Testosterone[Text Word]) OR TE[Text Word]) OR Nandrolone[Text Word]) OR Androgen[Text Word]) OR Testosterone[Text Word]) OR Dehydroepiandrosterone[Text Word]) OR Dihydrotestosterone[Text Word]) OR Anabolic Steroid[Text Word]) OR DHEA[Text Word]) OR DHT[Text Word]) OR 19-nortestosterone[Text Word]) OR Oxandrolone[Text Word]) AND ((((((((((((((Pulmonary Emphysema[MeSH Terms]) OR Emphysema[Text Word]) OR Pulmonary Disease, Chronic Obstructive[MeSH Terms]) OR COPD[Text Word]) OR Chronic Obstructive Pulmonary Disease[Text Word]) OR COAD[Text Word]) OR Chronic Obstructive Airway Disease[Text Word]) OR Chronic Obstructive Lung Disease[Text Word]) OR Airflow Obstruction[Text Word]) OR Lung Diseases, Obstructive[MeSH Terms]) OR Obstructive Lung[Text Word]) OR Obstructive Pulmonary[Text Word]) OR Bronchitis[Text Word]) OR Bronchitis[MeSH Terms])))) NOT Asthma[Text Word]) NOT Asthma[MeSH Terms]

1.2 For Embase

('sex hormone'/exp OR 'sex hormone' OR 'nandrolone'/exp OR nandrolone OR 'androgen'/exp OR androgen OR 'testosterone'/exp OR testosterone OR 'dehydroepiandrosterone'/exp OR dehydroepiandrosterone OR 'dihydrotestosterone'/exp OR dihydrotestosterone OR 'anabolic agent'/exp OR 'anabolic agent' OR 'oxandrolone'/exp OR oxandrolone) AND ('lung emphysema':ti,ab,kw OR 'chronic obstructive lung disease':ti,ab,kw OR 'airway obstruction':ti,ab,kw) NOT asthma:ti,ab,kw

1.3 For Cochrane library

ID Search Hits

#1 MeSH descriptor: [Testosterone] this term only

#2 MeSH descriptor: [Gonadal Steroid Hormones] this term only

#3 MeSH descriptor: [Nandrolone] this term only

#4 MeSH descriptor: [Dehydroepiandrosterone] this term only

#5 MeSH descriptor: [Dihydrotestosterone] this term only

#6 MeSH descriptor: [Testosterone Congeners] this term only

#7 MeSH descriptor: [Oxandrolone] this term only

#8 (Sex Steroid*):ti,ab,kw

#9 (Sex Hormone*):ti,ab,kw

#10 (Steroid Hormone*):ti,ab,kw

#11 (Gonadal Steroid*):ti,ab,kw

#12 (Androgen*):ti,ab,kw

#13 (DHEA):ti,ab,kw

#14 (DHT):ti,ab,kw

#15 #1 or #2 or #3 or #4 or #5 or #6 or #7 or #8 or #9 or #10 or #11 or #12 or #13 or #14

#16 MeSH descriptor: [Pulmonary Emphysema] this term only

#17 MeSH descriptor: [Emphysema] this term only

#18 MeSH descriptor: [Pulmonary Disease, Chronic Obstructive] this term only

#19 MeSH descriptor: [Lung Diseases, Obstructive] this term only

#20 MeSH descriptor: [Bronchitis] this term only

#21 #16 or #17 or #18 or #19 or #20

#22 #15 and #21 in Trials

1.4 For ClinicalTrials.gov

Advanced Search

Conditions: “Chronic Obstructive Pulmonary Disease”

Interventions: “drug: testosterone” / “drug: androgen” / “drug: dehydroepiandrosterone” / “drug: nandrolone” / “drug: oxandrolone”
